# Phosphodiesterase BpdE suppress bacterial proliferation by regulating *min* operon in *Brucella abortus*

**DOI:** 10.3389/fmicb.2026.1899258

**Published:** 2026-08-14

**Authors:** Jialu Chen, Zheng Dong, Wei Liu, Yaping Zu, Wei Liu, Dong Zhou, Yaping Jin, Aihua Wang

**Affiliations:** 1 College of Veterinary Medicine, Northwest A&F University, Yangling, Shaanxi, China; 2 Key Laboratory of Animal Biotechnology of the Ministry of Agriculture, Northwest A&F University, Yangling, Shaanxi, China; 3 State Key Laboratory for Diagnosis and Treatment of Severe Zoonotic Infectious Diseases, Key Laboratory of Zoonosis Research, Ministry of Education, Changchun, Jilin, China

**Keywords:** bacterial cell cycle, BpdE, *Brucella*, *minCDE*, phosphodiesterase

## Abstract

*Brucella* spp. are intracellular pathogens utilizing a unique, asymmetric cell cycle to survive within host macrophages. While the biogenesis of the replicative niche is well-studied, the intrinsic regulatory circuits that control the *Brucella* cell cycle remain incompletely understood. Phosphodiesterase (PDEs) modulates c-di-GMP levels and thereby contribute to the bacterial cell cycle. However, little is known about the role of PDEs in the cell cycle and mechanisms of *B. abortus*. In this study, we firstly demonstrate that BpdE is a high conserved protein across major *Brucella* species. Deletion of *bpdE* (Δ*bpdE*) significantly accelerated bacterial proliferation in vitro and enhanced intracellular survival within host macrophages. We found that Δ*bpdE* mutant exhibited an accumulation of DNA content compared to *B. abortus* A19 (WT) and complemented strain (CΔ*bpdE*). BpdE significantly affected the transcription of cell cycle regulators, including the *min* system. Additionally, we confirmed that the *minCDE* genes constitute a single operon in *B. abortus*. Furthermore, β-galactosidase reporter assays demonstrated that BpdE positively regulates the activity of the *minCDE* promoter. Taken together, we reported that the c-di-GMP phosphodiesterase BpdE represses bacterial proliferation by regulating *minCDE* operon in *B. abortus*. Our results provide a novel regulatory axis for bacterial proliferation and offer new insights into the intricate mechanisms of *Brucella* pathogenesis.

## 1 Introduction

*Brucella* is a Gram-negative, facultative intracellular pathogen that infects a wide range of hosts, including domesticated and wild terrestrial and marine mammals (Roop et al., 2021). The species primarily responsible for brucellosis are *B. abortus* (cattle), *B. melitensis* (goats and sheep), *B. ovis* (sheep), *B. suis* (pig), and *B. canis* (dog), with humans serving as accidental host (Whatmore and Foster, 2021). As a stealthy pathogen, *Brucella* can infect and replicate within various cell types, including macrophages, dendritic cells and trophoblasts (Carvalho et al., 2023). Throughout its evolution, *Brucella* has acquired the capability to modulate multiple cellular pathways to survive and replicate within host cells (Dugelay et al., 2025). However, while host-related factors are crucial for the biogenesis of the replicative *Brucella-*containing vacuole (rBCV), the regulatory mechanisms of the intrinsic bacterial cell cycle require further investigation.

In contrast to classical intracellular pathogens, *B. abortus* exhibits a distinctive cell cycle characterized by asymmetric cell division and cell polarity (De Bolle et al., 2015). Unipolar growth occurs at a single pole and the future division site, where new cell envelope components are incorporated (Van der Henst et al., 2013). The process is tightly associated with DNA replication. Transposon sequencing homology with *Escherichia coli* and *Caulobacter crescentus*, many genes are predicted to be involved in *B. abortus* cell division (Sternon et al., 2018). Emerging evidence highlights key functional components of this process, including those essential for DNA replication (such as the master regulator CtrA and the RepABC system) and cell division (such as the amidase DipM and the protease CdlP) (Roba et al., 2022).

Cyclic dimeric guanosine monophosphate (c-di-GMP) is a global nucleotide second messenger molecule in bacteria (Hengge, 2021). Phosphodiesterase (PDEs) containing EAL or HD-GYP domains degrade c-di-GMP into pGpG in a Mg^2+^/Mn^2+^-dependent manner (Jenal et al., 2017). Several bacteria utilize c-di-GMP to coordinate morphogenesis and developmental transitions. In *Caulobacter crescentus*, the PDEs PdeA and PleD direct polar morphogenesis and cell-cycle progression, while c-di-GMP modulate the activity of the cell cycle kinase CckA to initiate DNA replication (Abel et al., 2011; Lori et al., 2015; Wang et al., 2021). Similarly, in *Pseudomonas aeruginosa*, the PDE PA5017 (*pch*) is associated with bacterial population dynamics during the logarithmic phase; furthermore, heterogeneity in c-di-GMP levels has been observed during cell division and proliferation (Feng et al., 2024). However, it remains to be established whether PDE regulate cell proliferation and expression of cell division genes in *B. abortus.*

In this study, we exhibited a functional link between the PDE BpdE and bacterial proliferation in *B. abortus* A19. Our findings indicated that *RS_15270* (EAL domain-containing protein, designated BpdE) is a high conserved protein across major *Brucella* species. Interestingly, deletion of the *bpdE* gene (Δ*bpdE*) mutant significantly accelerated bacterial growth in vitro. Additionally, DNA content analysis confirmed that BpdE modulates the normal bacterial cell cycle. Furthermore, we examined nine genes related to cell cycle and found that the transcription levels of *minCDE* were significantly reduced in Δ*bpdE* compared to wild type (WT) strain. Finally, RT-PCR and β-galactosidase assays demonstrated that *minCDE* functions as an operon in *Brucella* and BpdE positively regulates its promoter activity. These findings enhance our understanding of PDEs signaling and the regulatory mechanisms of bacterial proliferation, providing novel insights into the regulatory network governing *Brucella* proliferation and intracellular survival.

## 2 Results

### 2.1 Conservation analysis of phosphodiesterase BpdE and construction of its deletion and complementation strains

**FIGURE 1:**
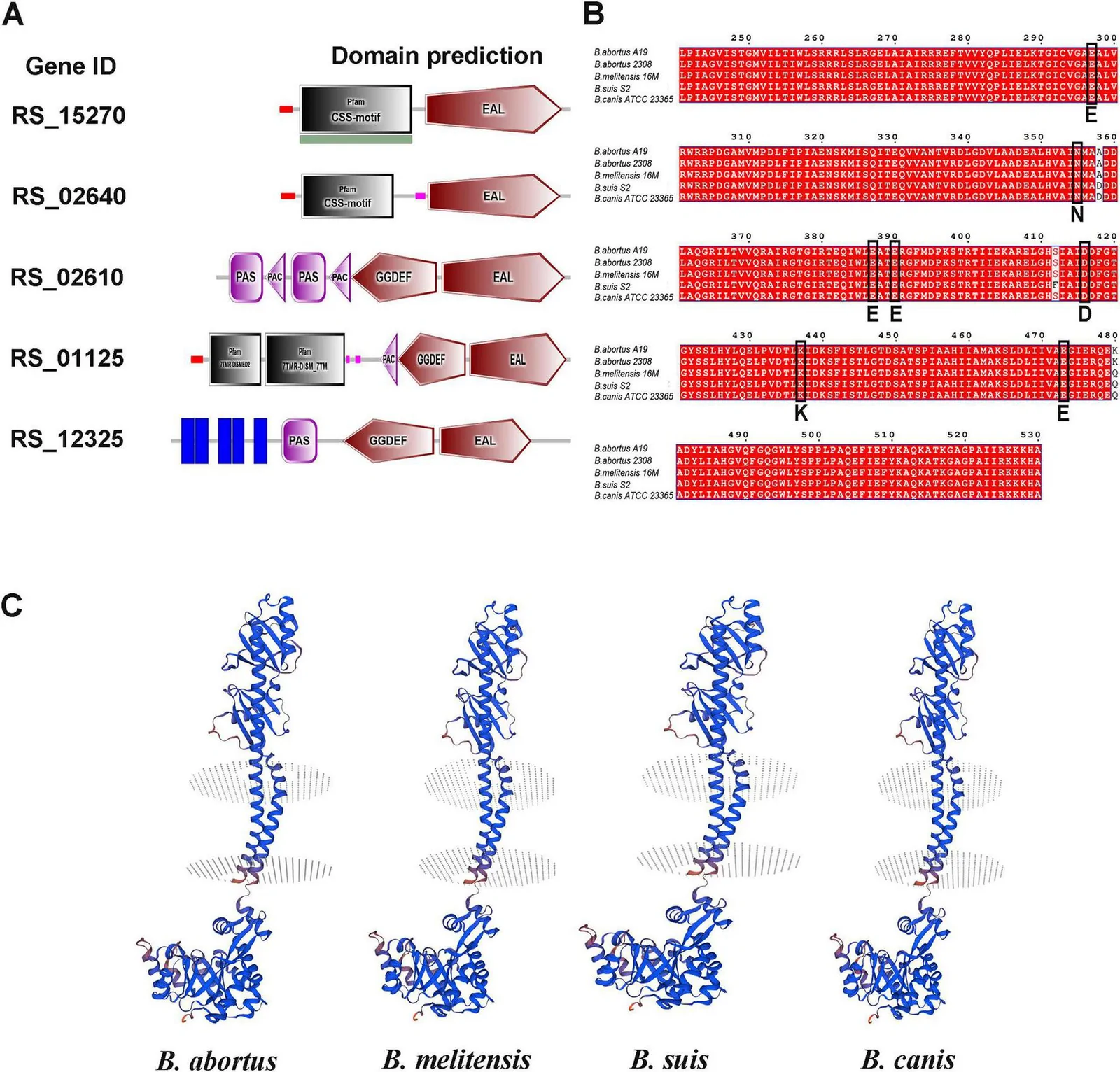
*Structural and sequence analysis of BpdE from B. abortus A19.* **(A)** Predicted EAL proteins in *B. abortus* A19. Domain architecture of EAL proteins created by SMART protein analysis tool highlights the EAL domains (red pentagon), PAS and PAC domains (purple boxes), Pfam CSS-motifs (black box), and predicted transmembrane helices (blue lines). **(B)** Sequence alignment of BpdE orthologs from four major *Brucella* species. The highly conserved “ENEEDKE” catalytic motif within the PDE domain is outlined in black boxes. **(C)** 3D structural models of BpdE and its orthologs from the four representative Brucella strains.

To define the role of RS15270 phosphodiesterase BpdE in *B. abortus* A19 pathogenesis, SMART domain analysis was first used to identify two key functional domains in BpdE (Figure 1A): an N-terminal region containing two transmembrane helices and C-terminal EAL domain (InterPro ID: IPR000154). The presence of the EAL domain, a well-characterized module for cyclic di-GMP (c-di-GMP) degradation, suggests BpdE functions as a phosphodiesterase involved in modulating intracellular c-di-GMP levels (Figure 1B). Furthermore, the 3D structural alignment revealed high conservation between BpdE and its homologs in major *Brucella* species (Figure 1C). These results have established a foundation for subsequent research on the role of BpdE in *Brucella*.

To investigate the function of BpdE, a *bpdE* deletion mutant (Δ*bpdE*) and its complemented strain (CΔ*bpdE*) were constructed via homologous recombination in *B. abortus* A19 (Supplementary Figures S1, S3). Sanger sequencing of the PCR product confirmed the junction and the complete deletion of *bpdE* (Supplementary Figure S2).

### 2.2 bpdE deletion significantly promoted the bacterial proliferation of *B. abortus*

**FIGURE 2:**
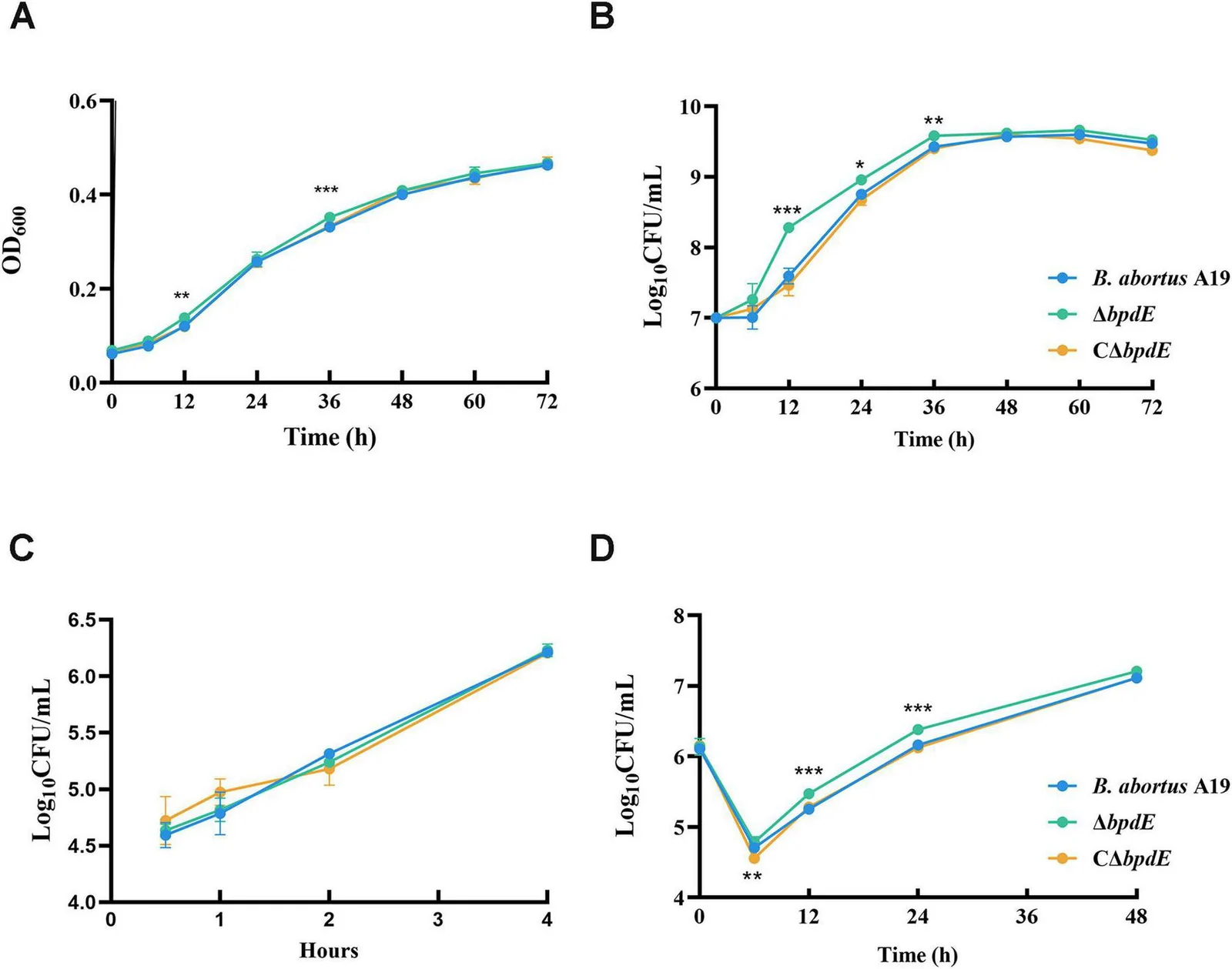
BpdE promoted the proliferation and intracellular survival of B. abortus A19. In vitro growth curves of wild-type *B. abortus* A19 and the ΔbpdE mutant strain over 72 h, as measured by OD_600_ **(A)** and CFU counts **(B)**. **(C)** Adhesion ability of ΔbpdE strain to RAW264.7 macrophages. **(D)** Intracellular proliferation of ΔbpdE strain within RAW264.7 macrophages monitored from 0 to 48 h post-infection. Data are presented as the mean ± SD (*n* = 3). Statistical significance was determined using two-way ANOVA followed by Tukey’s multiple-comparison test (ns, not significant; *P < 0.05; **P < 0.01; ***P < 0.001).

To investigate the role of BpdE in *Brucella* replication, we first evaluated the *in vitro* growth kinetics of *B. abortus* A19 (WT), Δ*bpdE* and CΔ*bpdE* over a 72 h period. Interestingly, during logarithmic growth phase, Δ*bpdE* exhibited significantly higher absorbance (OD_600_) and CFU counts compared to both the WT and CΔ*bpdE* (Figures 2a,b). Next, we assessed whether BpdE influences bacterial proliferation within macrophages. While no significant differences in adhesion to RAW 264.7 cells were observed among the three strains at 0.5, 1, 2, and 4 h post-infection (hpi) (Figure 2C), the Δ*bpdE* displayed a significantly increased intracellular bacterial load at 6, 12 and 24 hpi relative to the WT strain (Figure 2D). These results suggest that deletion of the *bpdE* gene accelerates the growth of *B. abortus* in vitro and enhances its intracellular proliferation in macrophages.

### 2.3 bpdE deletion significantly impacts the cell morphology and DNA content of *B. abortus*

**FIGURE 3:**
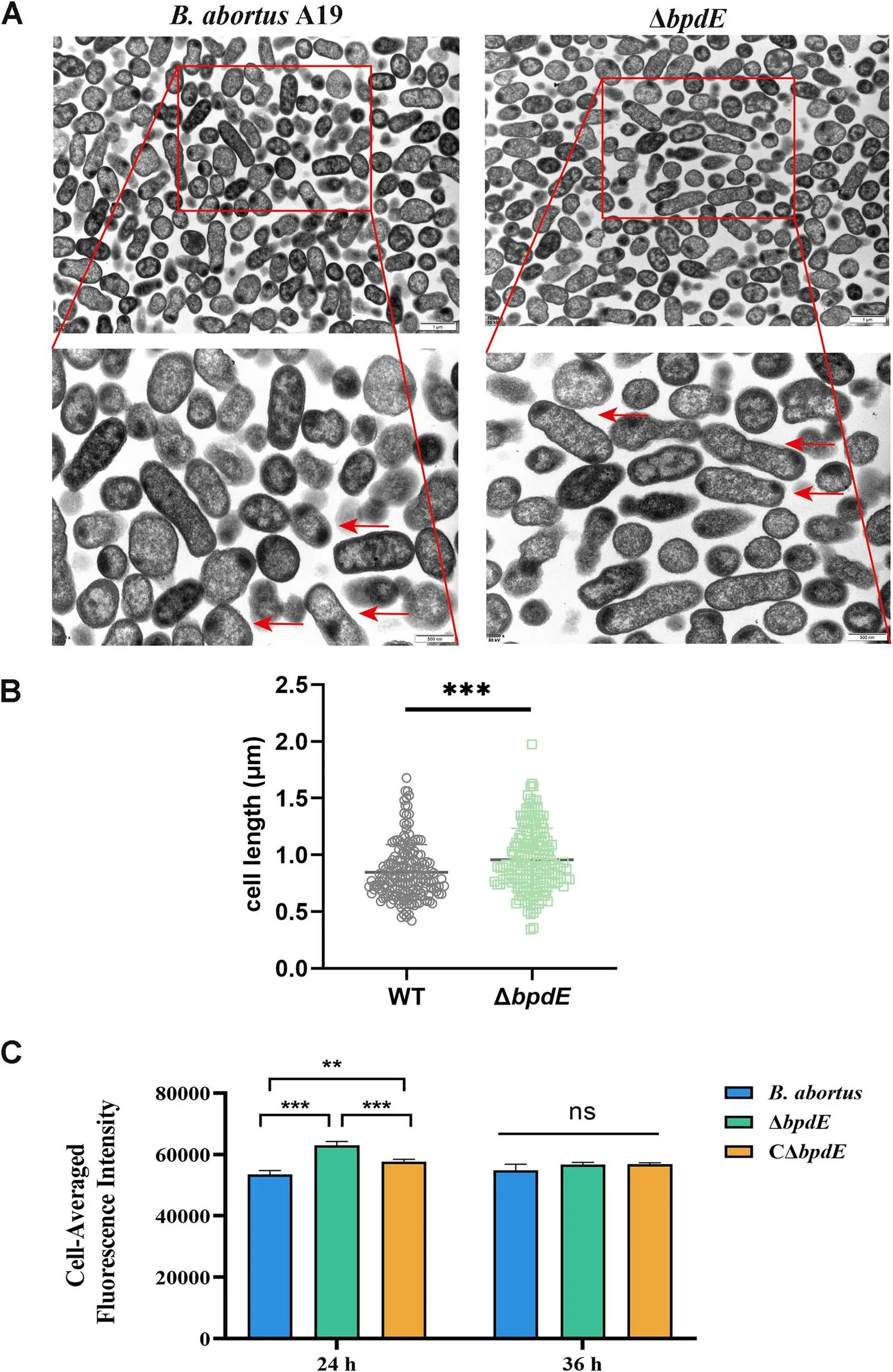
BpdE affects the cell morpholog and DNA content of B. abortus A19. **(A)** Transmission electron microscopy (TEM) analysis reveals altered cell morphology in the ΔbpdE compared to the WT strain. **(B)** Quantification of cell length using ImageJ (*n* = 180 cells from three biological replicates), analyzed by unpaired Student’s *t*-test. **(C)** Flow cytometric analysis of DNA content in *B. abortus* A19, ΔbpdE, and CΔbpdE strains. Bacterial cells were harvested at 24 h and 36 h post-incubation and stained with SYTOX Green. The DNA-associated fluorescence of 500,000 cells per sample was recorded to assess the distribution of DNA content. Data are presented as the mean ± SD (*n* = 3). Statistical significance was determined using two-way ANOVA followed by Tukey’s multiple-comparison test (ns, not significant; **P < 0.01; ***P < 0.001).

As with all living systems, bacterial cell proliferation requires the precise coordination of two fundamental cycles: DNA replication and cytokinesis (Meunier et al., 2021). Transmission electron microscopy (TEM) revealed morphological alterations in the Δ*bpdE* mutant (Figure 3A). Quantitative analysis of cell length demonstrated that the Δ*bpdE* group was significantly longer than the WT group (Figure 3B).

To quantify DNA synthesis dynamics, we utilized SYTOX Green, a well-established fluorescent probe for monitoring DNA replication (Deghelt et al., 2014). At the 24 h time point, the intracellular DNA content of Δ*bpdE* was significantly higher than that of the WT strain. However, by 36 h, the DNA content in the mutant had not significantly changed compared with that of WT and CΔ*bpdE* (Figure 3C). These results indicate that the deletion of *bpdE* disrupts the fine-tuned regulation of DNA synthesis during the normal bacterial cell cycle.

### 2.4 Deletion bpdE of significantly decreased minCDE mRNA levels

**FIGURE 4:**
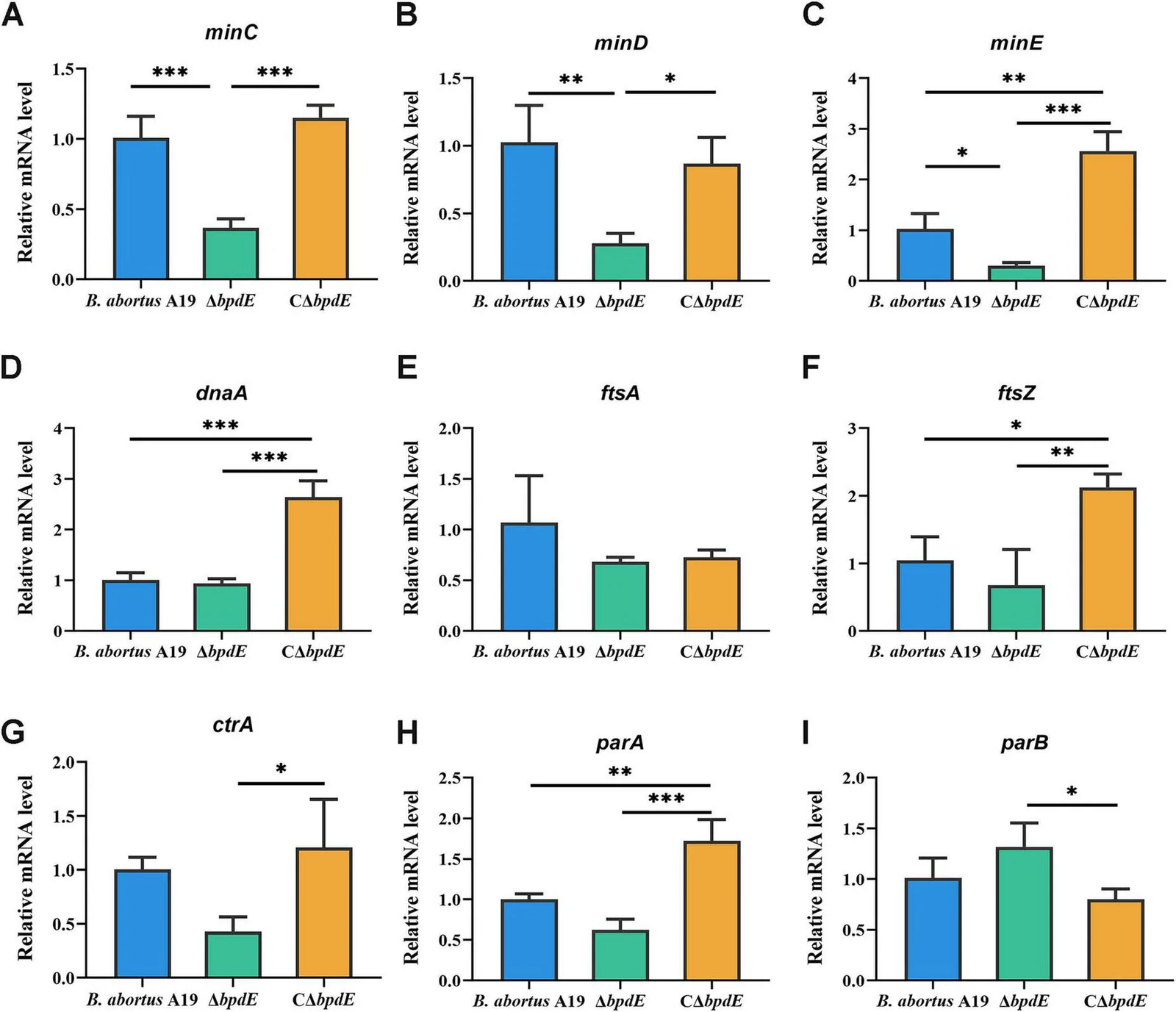
BpdE regulates the transcription of genes involved in cell division and DNA replication. Relative mRNA expression levels of minC **(A)**, minD **(B)**, minE **(C)**, dnaA **(D)**, ftsA **(E)**, ftsZ **(F)**, ctrA **(G)**, parA **(H)**, and parB **(I)** were determined by qRT-PCR in *B. abortus* A19, ΔbpdE, and CΔbpdE strains. Data presented means ± SD (*n* = 3). Statistical significance was analyzed using an unpaired Student’s *t*-test (*P < 0.05; **P < 0.01; ***P < 0.001).

A common feature in bacteria is that DNA replication and segregation are two largely overlapping events, both of which are tightly regulated by a complex network involving multiple genes (Meunier et al., 2021). We selected a total of 9 genes linked to the bacterial proliferation network for quantitative real-time PCR (RT-qPCR) validation. Our results showed that Δ*bpdE* strain exhibited significantly reduced transcriptional levels of the *minCDE* system (septum site-determining proteins MinC/D and cell division topological specificity factor, MinE) compared to WT and CΔ*bpdE* strains (Figures 4A–C). However, Δ*bpdE* strain showed no significant changes in expression levels compared to WT in *dnaA* (chromosomal replication initiator protein, DnaA), *ftsA/Z* (cell division proteins, FtsA/Z), *ctrA* (cell cycle two-component system response regulator, CtrA) and *DR186_RS10410*/*RS10405* (ParA and ParB family proteins) (Figures 4D–I). These findings suggest that BpdE regulates the transcription of the *minCDE* system in *B. abortus*.

### 2.5 minCDE genes constitute a single operon in *B. abortus*

**FIGURE 5:**
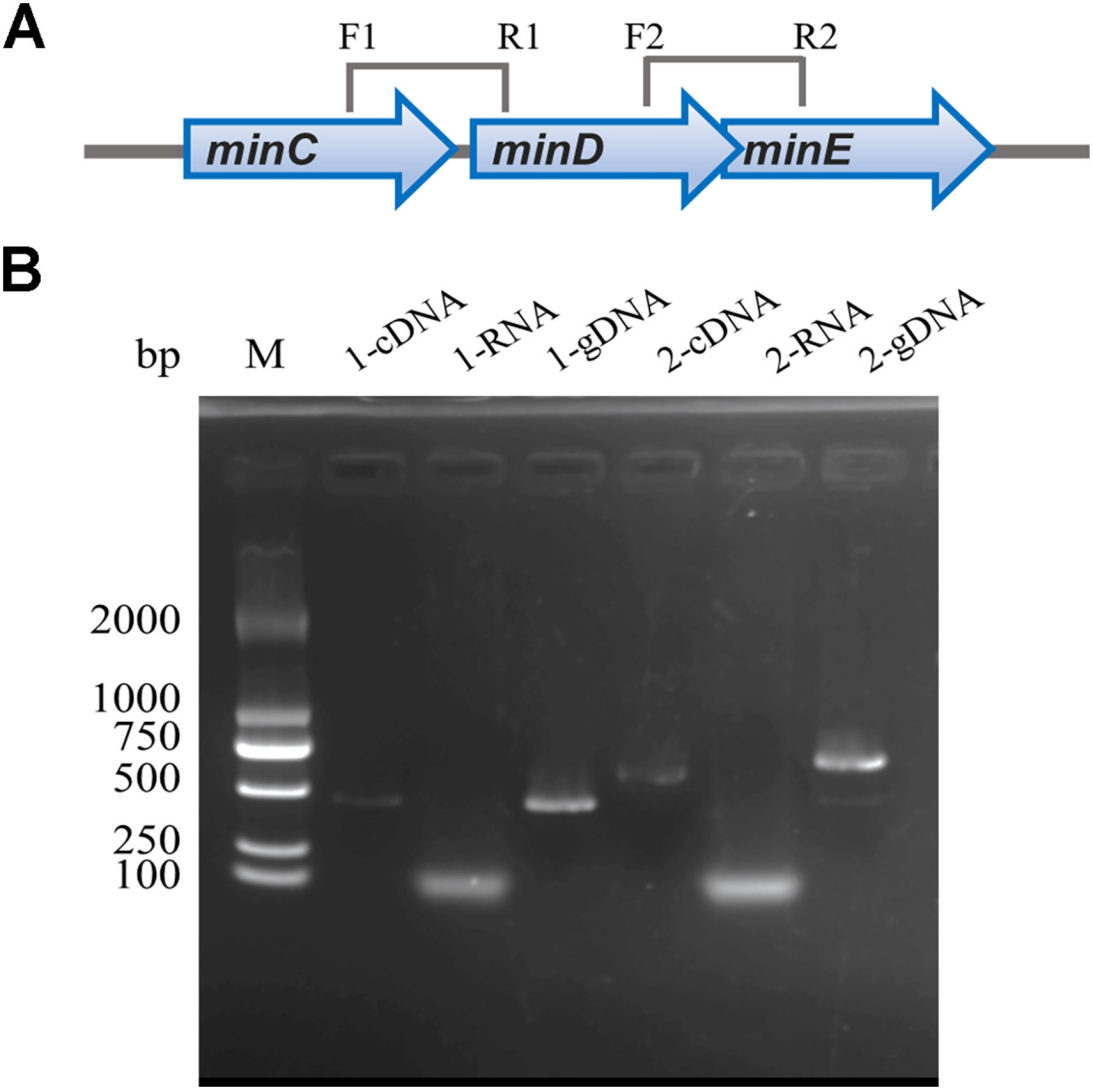
Co-transcription analysis of the minCDE gene cluster in B. abortus A19. **(A)** Schematic organization of the minCDE locus. Blue arrows indicate the orientation and relative positions of minC, minD, and minE genes. Gray lines (F1/R1 and F2/R2) represent the primer sets spanning the intergenic regions used to amplify fragments 1 and 2, respectively. **(B)** Reverse transcription-PCR verification of minCDE co-transcription. PCR amplification of fragments 1 and 2 was performed using cDNA, genomic DNA (gDNA, positive control) and total RNA (negative control) as templates. Lane M: DNA marker.

To elucidate the regulatory mechanism of BpdE on the *minCDE system*, we first determined its transcriptional architecture. The genetic organization of the *minCDE* locus and the reverse transcription-PCR (RT-PCR) strategy for amplifying overlapping regions (F1/R1 and F2/R2) are shown in schematic (Figure 5A). Using cDNA, gDNA (positive control), and RNA (negative control) as templates, specific PCR products of identical sizes were amplified from both cDNA and gDNA, whereas no products were detected in the RNA controls (Figure 5B). These findings demonstrate that *minCDE* is co-transcribed as a single operon from a common promoter in *B. abortus*.

### 2.6 Phosphodiesterase BpdE regulates the minCDE promoter in *B. abortus*

**FIGURE 6:**
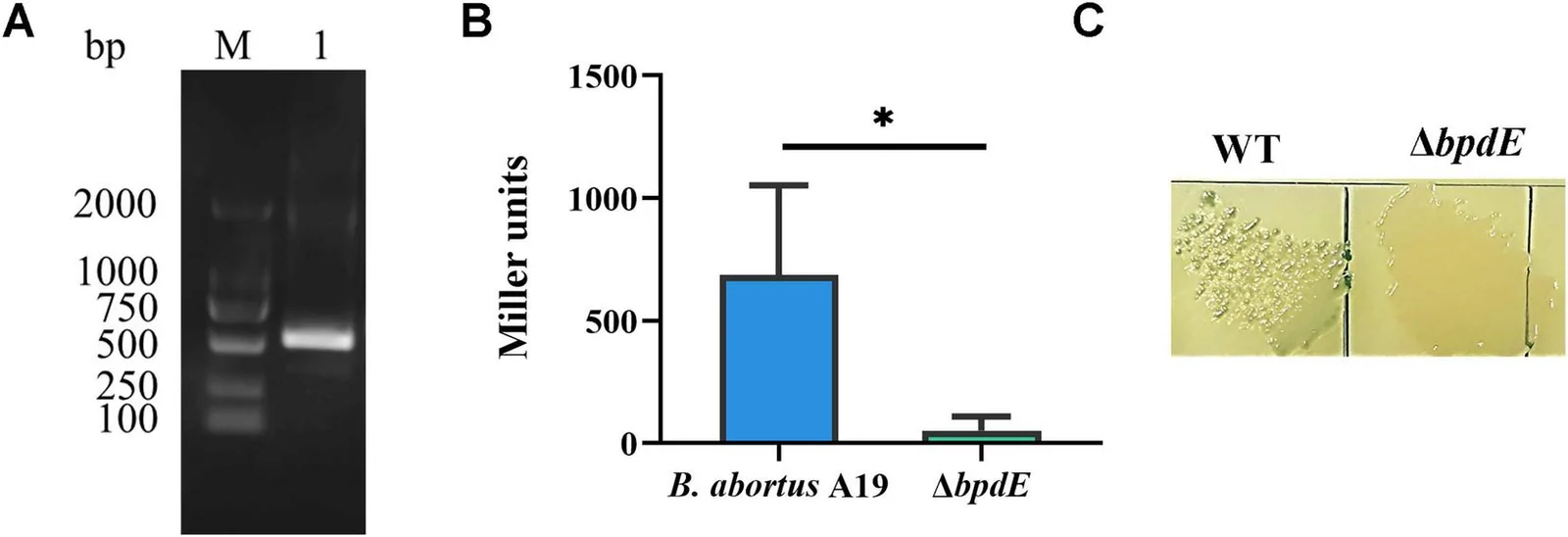
BpdE positively regulated the minCDE promoter in B. abortus A19. **(A)** PCR amplification of the minCDE promoter region (approximately 500 bp). Lane M: DNA marker; Lane 1: minCDE promoter fragment. **(B)** β-galactosidase activity of minCDE promoter reporter fusion in WT and ΔbpdE strains. **(C)** Blue-white screening of minCDE promoter between WT and ΔbpdE strains. Data presented means ± SD (*n* = 3). Statistical significance was determined by an unpaired Student’s *t*-test (*P < 0.05).

To functionally validate the regulation of *minCDE* transcription, we evaluated its promoter activity using a β-galactosidase reporter assay. First, a 500-bp region upstream of the *minC* gene was PCR-amplified (Figure 6A) and used to construct a promoter-reporter system. The WT and Δ*bpdE* strains harboring this construct were designated as WT-*minC*p and Δ*bpdE*-*minC*p, respectively. Quantitative analysis revealed that the β-galactosidase (Miller) activity driven by the *minCDE* promoter was significantly reduced in Δ*bpdE* mutant compared to WT strain (Figures 6B,C). Collectively, these findings demonstrate that the phosphodiesterase BpdE positively regulates the activity of the *minCDE* promoter in *B. abortus*.

## 3 Discussion

*Brucella abortus* is a Gram-negative, facultative intracellular pathogen responsible for abortion and sterility in cattle, as well as a debilitating febrile illness known as Malta fever in humans (Fiebig et al., 2021). Successful infection relies heavily on the strict regulation of the bacterial cell cycle in response to host-derived stresses (Verbeke et al., 2023). As a member of the *α-proteobacteria*, *B. abortus* regulate the replication of a complex genome during infection (Fiebig et al., 2021). The large circular chromosome (Chr I, 2.1 Mb) utilizes a ParAB segregation system with three centromere-like *parS* sites, whereas the smaller chromid (Chr II, 1.2 Mb) is governed by a RepABC replication system (Pinto et al., 2012; Huang et al., 2023) Upon internalization by host cells, such as HeLa cells and RAW 264.7 macrophages, *B. abortus* typically undergoes a prolonged G1-phase arrest for up to 8 h (Deghelt et al., 2014) Elucidating how *Brucella* coordinates the cell cycle and DNA replication is crucial for understanding the mechanisms of the infectious process itself. Here, we characterized BpdE (an EAL domain contain protein), which exhibits high conservatively across four major *Brucella* species. Deletion of *bpdE* (Δ*bpdE*) affects cell proliferation, intracellular survival, cell morphology, and the DNA content. Importantly, we found that Δ*bpdE* reduced the transcription of cell division associated genes. Furthermore, BpdE positive regulate *minCDE* promoter. In conclusion, BpdE contributes to bacterial proliferation in *B. abortus*.

The ubiquitous bacterial second messenger, cyclic di-GMP (c-di-GMP), governs a range of cellular behaviors, including motility, adhesion, and organelle biogenesis (Christen et al., 2010). Recent findings have further demonstrated that the spatiotemporal deployment of c-di-GMP metabolizing enzymes profoundly impacts bacterial polar division, thereby minimizing phenotypic heterogeneity and ensuring proper cell cycle progression (Pérez-Burgos et al., 2024). Consistent with this paradigm, deletion of the phosphodiesterase BpdE altered the growth kinetics and division phenotypes of *B. abortus*, indicating that BpdE contributes to coordinating the *Brucella* cell cycle. In the model organism *Caulobacter crescentus*, fluctuations in intracellular c-di-GMP concentrations regulate the transcription and polarization of flagellar regulator TipF, an event that is tightly coupled to the G1-to-S transition (Davis et al., 2013). Although *Brucella* is canonically considered a non-motile pathogen, its genome encodes a highly conserved, yet cryptic, flagellar apparatus that has been shown to be essential for full virulence (Li et al., 2012; Coloma-Rivero et al., 2020; Zhai et al., 2024). This parallel presents a fascinating avenue for future research. It is highly plausible that the BpdE-mediated c-di-GMP signaling network intersects with these cryptic flagellar components to regulate cell cycle progression and virulence. Future investigations exploring the crosstalk between c-di-GMP homeostasis, flagellar gene expression, and the cell division machinery will be instrumental in deciphering the unique pathogenic strategies of *Brucella*.

Unlike eukaryotes, many bacteria, including members of the *α-proteobacteria* do not possess a distinct, checkpoint-gated cell cycle (Panis et al., 2015). The commitment to genome duplication, which occupies a substantial portion of the cell cycle, represents a pivotal and irreversible event that must proceed to completion once initiated (Sakiyama et al., 2017; Reyes-Lamothe and Sherratt, 2019). Our finding that the DNA content in the Δ*bpdE* mutant was significantly higher than in the WT and complemented strains suggests that the loss of BpdE may accelerated entry into the DNA replication period. A comparative analysis across five *alphaproteobacterial* orders revealed that the spatial distribution of c-di-GMP-metabolizing enzymes is potentially linked to temporary gene copy number imbalances during the cell cycle (Koppenhöfer and Lang, 2022). In *Vibrio cholerae*, such imbalances have been demonstrated to significantly alter chromosomal replication dynamics and genome-wide transcription (Soler-Bistué et al., 2020). Whether gene copy number imbalances drive the observed DNA shifts remains to be experimentally confirmed.

In the model organism *Caulobacter crescentus*, which also undergoes asymmetric division, the cell cycle is governed by regulators CtrA, DnaA, ParA, ParB and MucR (Fröhlich and Velasco Gomariz, 2021; McLean and Le, 2023) Intriguingly, while these master regulators showed no significant transcriptional changes in Δ*bpdE* mutant, we observed a marked downregulation of the *minCDE* operon. In *Escherichia coli*, the *min* system (*minC*, *minD*, and *minE*) acts as a spatial regulator that restricts FtsZ-ring assembly to the mid-cell, thereby preventing aberrant polar division (LaBreck et al., 2021; Wagner et al., 2022). The specific impact of *bpdE* deletion on the *min* system, rather than the global *ctrA* pathway, indicates that BpdE-mediated growth control is channeled through the precise spatial regulation of the division machinery. Furthermore, recent evidence in *Xanthomonas* has linked the *minCDE* system to the coordination of Type III secretion systems (T3SS) and overall virulence (Yan et al., 2022). Given that *Brucella* must strictly tune its proliferation to facilitate intracellular survival, the BpdE–*minCDE* axis likely represents a sophisticated mechanism for balancing replication kinetics with host immune evasion. Future studies characterizing the direct interaction between the Min system and FtsZ filaments in *Brucella* will be essential to fully decipher how this pathogen orchestrates its replicative cycle during infection.

In summary, our findings confirm that the phosphodiesterase BpdE plays a key role in the proliferation of *B. abortus*. The deletion of *bpdE* results in increased bacterial proliferation and intracellular survival in macrophages. Furthermore, our results demonstrated that *bpdE* deletion alters cell morphology and DNA content. Moreover, our results reveal that deletion of *bpdE* reduces the mRNA levels of the *min* system by regulating the activity of its operon promoter. These findings reveal a novel cell cycle mechanism mediated by PDE enzyme signaling in *Brucella*.

## 4 Materials and methods

### 4.1 Bacteria, culture conditions

The *Brucella abortus* A19 (CVCC70202) was obtained from Shaanxi Veterinary Drug Inspection Institute and cultured in tryptic soy broth (TSB, Sigma-Aldrich, United States) or tryptic soy agar (TSA, Sigma-Aldrich, United States) at 37°C and 180 rpm. Experiments using live *B. abortus* A19 were performed in biosafety level 2 facility. *Escherichia coli* DH5α was cultured in LB broth and on LA plates for plasmid cloning.

### 4.2 BpdE conservation comparisons

The conserved structural domains and transmembrane helices of *B. abortus* A19 BpdE were analyzed and annotated using the SMART^1^ and TMHMM (ver 2.0), respectively. To evaluate the conservation of BpdE among major *Brucella* species, multiple sequence alignment was performed using the ClustalW algorithm (ver 2.1). The analyzed sequences included those from *B. abortus* (WP_002966246.1), *B. melitensis* (WP_004686859.1), *B. canis* (WP_004690097.1) and *B. suis* (WP_006191802.1). Furthermore, the three-dimensional (3D) structure of BpdE was predicted via homology modeling using the SWISS-MODEL (SWISS-MODEL) server.

### 4.3 Construction of bpdE mutant and complemented strain

The *bpdE* deletion of *B. abortus* A19 was generated via allelic exchange. Briefly, the upstream and downstream homologous fragments of the *bpdE* gene were amplified and used as templates for overlap PCR. The pUC19-kan-SacB vector was double-digested with *Hind*III and *Sal*I restriction enzymes. The target overlapping DNA fragment was purified using a DNA recovery kit (Tiangen, DP209, China), and inserted into the digested plasmid using a One-step Cloning kit (Vazyme, C112, China). The resulting suicide plasmids were electroporated into competent *B. abortus* A19 cells. The first homologous recombination event was selected on TSA containing kanamycin (50 μg/mL), and the second recombination was selected on TSA containing sucrose (8%). The deletion strains (Δ*bpdE*) were verified by PCR using primers *bpdE*-I-F/R.

To complement the mutation, the *bpdE* gene, including its native promoter, was amplified by PCR. The pBBR-amp-flag shuttle vector (maintained in our laboratory), which carries an ampicillin resistance gene and a C-terminal Flag-tag, was digested with *Hind*III. The recombinant plasmid was electroporated into competent Δ*bpdE* cells, and the complemented strain was selected on TSA containing ampicillin (100 μg/mL). The successful construction of the complemented strain (CΔ*bpdE*) was confirmed by Western blot using an anti-DYKDDDDK Tag antibody (1:2,000, HT201, TRANS, China) and an anti-*Brucella* Omp16 mAb (1:1,000) (Zhai et al., 2025) as a loading control, as well as by qRT-PCR using primers RT-*bpdE*-F/R. All plasmids and primers utilized are listed in Supplementary Table S1.

### 4.4 Determination of bacterial growth phenotype

The *B. abortus* A19, Δ*bpdE*, and CΔ*bpdE* strains were cultured to the exponential phase and diluted to a density of 1 × 10^7^ CFU/mL. A 100 μL aliquot of each bacterial suspension was inoculated into 20 mL of TSB medium and incubated at 37°C with shaking (180 rpm). At specified intervals (0, 12, 24, 36, 48, 60, and 72 h), 100 μL samples were withdrawn to determine viable counts via plate assay, cell by plate assay, while the OD_600_ was measured using a microplate reader (TECAN, Switzerland).

### 4.5 Macrophage infection

The murine macrophage cell line RAW 264.7 used in this study was purchased from the Cell Bank of the Chinese Academy of Sciences (Shanghai, China). Cells were maintained in high-glucose DMEM (Beyotime, C2701, China) supplemented with 5% fetal bovine serum (Gibco, A5256701, United States) and cultured at 37°C under an atmosphere of 5% CO_2_.

Adhesion assay: RAW 264.7 cells were seeded in 24-well plates (2 × 10^5^ cells per well) and infected with *B. abortus* A19 and its derivatives at a multiplicity of infection (MOI) of 100:1. The time of bacterial addition was designated as 0 h. At 0.5, 1, 2, and 4 h post-infection, cells were washed twice with PBS to remove non-adherent bacteria and lysed with 0.5 mL of 0.5% Triton X-100 in PBS at 37 °C for 10 min. Lysates were serially diluted 10-fold and plated onto TSA for colony counting.

Intracellular survival assay: RAW 264.7 cells were seeded in 24-well plates (2 × 10^5^ cells per well) and infected with *B. abortus* A19 and its derivatives for 4 h at an MOI of 100:1. Cells were incubated with 50 μg/mL gentamicin for 1 h to eliminate extracellular bacteria and maintained in 25 μg/mL gentamicin. At indicated time points, RAW 264.7 cells were lysed in 0.5 mL of PBS containing 0.5% Triton X-100, and the lysates were plated on TSA to determine intracellular CFU counts.

### 4.6 Transmission electron microscope

*B. abortus* A19 and Δ*bpdE* strains were inoculated at a 1:100 ratio into 20 mL of TSB in 50 mL centrifuge tubes. The cultures were incubated at 37°C with shaking at 180 rpm until reaching the logarithmic growth phase. Cells were harvested by centrifugation at 5,000 rpm for 10 min at 4°C, and the supernatants were discarded. The bacterial pellets were resuspended and fixed in 1 mL of 2.5% glutaraldehyde for 4 h at room temperature. The samples were then washed three times with ultrapure water, post-fixed with 1% osmium tetroxide for 1 h, and washed again three times with ultrapure water. Subsequently, the pellets were dehydrated through a graded ethanol series (30, 50, 70, 90, and 100%) for approximately 15 min at each concentration. Finally, the samples were embedded in Epon-812, ultra-thin sectioned (90 nm), and stained with uranyl acetate and lead citrate. Micrographs were captured using a JEM-1400-FLASH Transmission Electron Microscope (JEOL, Japan).

### 4.7 Analysis of DNA content

DNA content was measured as previously described (Deghelt et al., 2014). Briefly, *B. abortus* A19, Δ*bpdE* and CΔ*bpdE* strains harvested at 24 h and 36 h were fixed in ice-cold 77% ethanol and treated with 0.1 mg/mL of RNAse A (Thermo, EN0531, United States). Cells were then stained with SYTOX Green (Beyotime, C1186, China) and analyzed using a NovoCyte flow cytometer (Agilent Technologies, CA, United States). For each sample, the DNA-associated fluorescence of 500,000 cells was recorded and processed via NovoExpress software.

### 4.8 Gene expression analysis by quantitative real-time PCR (qRT-PCR)

The total RNA was extracted from cultures grown to the exponential phase using RNAiso plus (Takara, 9109). The first-strand cDNA was synthesized using the EvoM-MLVRT Mix Kit (AG11728, China). Gene-specific primers were designed targeting bacterial proliferation network (Supplementary Table S2). The 16S rRNA gene was used as an internal reference. qRT-PCR was performed using SYBR Green Premix (AG11701, China) on a CFX96 Real-Time PCR Detection System (Bio-Rad). Reactions were run in triplicate, and relative gene expression was calculated using the 2^–ΔΔCt^ method. All experiments were performed in biological triplicate.

### 4.9 Reverse transcription-PCR (RT-PCR) analysis for minCDE operon

Primers RT-F and RT-R (Supplementary Table S2) spanning the *minC*, *minD* and *minE* genes were designed to examine their co-transcription. Total RNA was extracted from *B. abortus* A19 at the exponential phase and reverse-transcribed into cDNA, as described above. The co-transcription of *minCDE* was verified by PCR amplification using the synthesized cDNA as a template, with genomic DNA serving as a control.

### 4.10 β-galactosidase assays

β-galactosidase activity was measured in triplicate according to previously established methods (Liu et al., 2026). The promoter fragment of *minCDE* was amplified via PCR from *B. abortus* A19 genomic DNA using the primers listed in Supplementary Table S2. The resulting minCp-lacZ fusion plasmids were electroporated into competent *B. abortus* A19 and Δ*bpdE* cells (WT-minCp and Δ*bpdE*-minCp). Transformants were cultured at 37°C, and single colonies were inoculated into 20 mL TSB and growth to logarithmic phase. Cells were harvested by centrifugation, washed with ice-clod PBS, and subsequently subjected to β-galactosidase assay.

### 4.11 Statistical analysis

All experiments were performed in triplicate, and data are presented as the mean ± standard deviation (SD). Statistical analyses were performed using two-way ANOVA followed by Tukey’s post-test (GraphPad Prism 9, San Diego, CA, United States) unless otherwise indicated. Statistical significance was defined as follows: *P* > 0.05 (ns, not significant); *P* < 0.05 (*); *P* < 0.01 (**) and *P* < 0.001 (***).

## Data Availability

The original contributions presented in the study are included in the article/Supplementary material, further inquiries can be directed to the corresponding author.

## Footnotes

1
https://smart.embl.de

## References

[B1] Abel S. Chien P. Wassmann P. Schirmer T. Kaever V. Laub M. T. et al. (2011). Regulatory cohesion of cell cycle and cell differentiation through interlinked phosphorylation and second messenger networks. *Mol. Cell* 43 550–560. 10.1016/j.molcel.2011.07.018 21855795 PMC3298681

[B2] Carvalho T. P. Silva L. A. D. Castanheira T. L. L. Souza T. D. Paixão T. A. D. Lazaro-Anton L. et al. (2023). Cell and tissue tropism of brucella spp. *Infect. Immun*. 91:e0006223. 10.1128/iai.00062-23 37129522 PMC10187126

[B3] Christen M. Kulasekara H. D. Christen B. Kulasekara B. R. Hoffman L. R. Miller S. I. (2010). Asymmetrical distribution of the second messenger c-di-GMP upon bacterial cell division. *Science* 328 1295–1297. 10.1126/science.1188658 20522779 PMC3906730

[B4] Coloma-Rivero R. F. Gómez L. Alvarez F. Saitz W. Del Canto F. Céspedes S. et al. (2020). The role of the flagellar protein flgj in the virulence of brucella abortus. *Front. Cell Infect. Microbiol*. 10:178. 10.3389/fcimb.2020.00178 32411617 PMC7198779

[B5] Davis N. J. Cohen Y. Sanselicio S. Fumeaux C. Ozaki S. Luciano J. et al. (2013). De- and repolarization mechanism of flagellar morphogenesis during a bacterial cell cycle. *Genes Dev*. 27 2049–2062. 10.1101/gad.222679.113 24065770 PMC3792480

[B6] De Bolle X. Crosson S. Matroule J. Y. Letesson J. J. (2015). Brucella abortus cell cycle and infection are coordinated. *Trends Microbiol*. 23 812–821. 10.1016/j.tim.2015.09.007 26497941 PMC8800490

[B7] Deghelt M. Mullier C. Sternon J. F. Francis N. Laloux G. Dotreppe D. et al. (2014). G1-arrested newborn cells are the predominant infectious form of the pathogen brucella abortus. *Nat. Commun*. 5 4366. 10.1038/ncomms5366 25006695 PMC4104442

[B8] Dugelay C. Celli J. Terradot L. (2025). The brucella type IV secretion system and effector proteins. *Microlife*. 6:uqaf036. 10.1093/femsml/uqaf036 41358267 PMC12676571

[B9] Feng Q. Zhou J. Zhang L. Fu Y. Yang L. (2024). Insights into the molecular basis of c-di-GMP signalling in *Pseudomonas aeruginosa*. *Crit. Rev. Microbiol*. 50 20–38. 10.1080/1040841X.2022.2154140 36539391

[B10] Fiebig A. Vrentas C. E. Le T. Huebner M. Boggiatto P. M. Olsen S. C. et al. (2021). Quantification of brucella abortus population structure in a natural host. *Proc. Natl. Acad Sci. USA* 118:e2023500118. 10.1073/pnas.2023500118 33688053 PMC7980399

[B11] Fröhlich K. S. Velasco Gomariz M. (2021). RNA-controlled regulation in caulobacter crescentus. *Curr. Opin. Microbiol*. 60 1–7. 10.1016/j.mib.2021.01.002 33529919

[B12] Hengge R. (2021). High-specificity local and global c-di-GMP signaling. *Trends Microbiol*. 29 993–1003. 10.1016/j.tim.2021.02.003 33640237

[B13] Huang Y. F. Liu L. Wang F. Yuan X. W. Chen H. C. Liu Z. F. (2023). High-resolution 3d genome map of brucella chromosomes in exponential and stationary phases. *Microbiol. Spectr*. 11:e0429022. 10.1128/spectrum.04290-22 36847551 PMC10100373

[B14] Jenal U. Reinders A. Lori C. (2017). Cyclic di-GMP: Second messenger extraordinaire. *Nat. Rev. Microbiol*. 15 271–284. 10.1038/nrmicro.2016.190 28163311

[B15] Koppenhöfer S. Lang A. S. (2022). Patterns of abundance, chromosomal localization, and domain organization among c-di-GMP-metabolizing genes revealed by comparative genomics of five alpha*proteobacteria*l orders. *BMC Genomics*. 23:834. 10.1186/s12864-022-09072-9 36522693 PMC9756655

[B16] LaBreck C. J. Trebino C. E. Ferreira C. N. Morrison J. J. DiBiasio E. C. Conti J. et al. (2021). Degradation of MIND oscillator complexes by *Escherichia coli* ClpXP. *J. Biol. Chem*. 296:100162. 10.1074/jbc.RA120.013866 33288679 PMC7857489

[B17] Li X. Xu J. Xie Y. Qiu Y. Fu S. Yuan X. et al. (2012). Vaccination with recombinant flagellar proteins FlgJ and FliN induce protection against Brucella abortus 544 infection in BALB/c mice. *Vet. Microbiol*. 161 137–144. 10.1016/j.vetmic.2012.07.016 22854331

[B18] Liu X. Cui C. Wang P. Wang H. Zhai Y. Yang Y. et al. (2026). (P)ppGpp synthase Rsh promotes persister cells formation by regulating sulfur metabolism mediated by sulfate reductase CysI in Brucella abortus. *Vet. Microbiol*. 315:110949. 10.1016/j.vetmic.2026.110949 41734519

[B19] Lori C. Ozaki S. Steiner S. Böhm R. Abel S. Dubey B. N. et al. (2015). Cyclic di-GMP acts as a cell cycle oscillator to drive chromosome replication. *Nature* 523 236–239. 10.1038/nature14473 25945741

[B20] McLean T. C. Le T. B. (2023). CTP switches in ParABS-mediated bacterial chromosome segregation and beyond. *Curr. Opin. Microbiol*. 73:102289. 10.1016/j.mib.2023.102289 36871427

[B21] Meunier A. Cornet F. Campos M. (2021). Bacterial cell proliferation: From molecules to cells. *FEMS Microbiol. Rev*. 45:fuaa046. 10.1093/femsre/fuaa046 32990752 PMC7794046

[B22] Panis G. Murray S. R. Viollier P. H. (2015). Versatility of global transcriptional regulators in alpha-*Proteobacteria*: From essential cell cycle control to ancillary functions. *FEMS Microbiol. Rev*. 39 120–133. 10.1093/femsre/fuu002 25793963

[B23] Pérez-Burgos M. Herfurth M. Kaczmarczyk A. Harms A. Huber K. Jenal U. et al. (2024). A deterministic, c-di-GMP-dependent program ensures the generation of phenotypically similar, symmetric daughter cells during cytokinesis. *Nat. Commun*. 15:6014. 10.1038/s41467-024-50444-4 39019889 PMC11255338

[B24] Pinto U. M. Pappas K. M. Winans S. C. (2012). The ABCs of plasmid replication and segregation. *Nat Rev Microbiol*. 10 755–765. 10.1038/nrmicro2882 23070556

[B25] Reyes-Lamothe R. Sherratt D. J. (2019). The bacterial cell cycle, chromosome inheritance and cell growth. *Nat. Rev. Microbiol*. 17 467–478. 10.1038/s41579-019-0212-7 31164753

[B26] Roba A. Carlier E. Godessart P. Naili C. De Bolle X. (2022). Histidine auxotroph mutant is defective for cell separation and allows the identification of crucial factors for cell division in brucella abortus. *Mol. Microbiol*. 118 145–154. 10.1111/mmi.14956 35748337

[B27] Roop R. M. Barton I. S. Hopersberger D. Martin D. W. (2021). Uncovering the hidden credentials of brucella virulence. *Microbiol. Mol. Biol. Rev*. 85 e21–e19. 10.1128/MMBR.00021-19 33568459 PMC8549849

[B28] Sakiyama Y. Kasho K. Noguchi Y. Kawakami H. Katayama T. (2017). Regulatory dynamics in the ternary DnaA complex for initiation of chromosomal replication in *Escherichia coli*. *Nucleic. Acids Res*. 45 12354–12373. 10.1093/nar/gkx914 29040689 PMC5716108

[B29] Soler-Bistué A. Aguilar-Pierlé S. Garcia-Garcerá M. Val M. E. Sismeiro O. Varet H. et al. (2020). Macromolecular crowding links ribosomal protein gene dosage to growth rate in *Vibrio cholerae*. *BMC Biol*. 18:43. 10.1186/s12915-020-00777-5 32349767 PMC7191768

[B30] Sternon J. F. Godessart P. Gonçalves de Freitas R. Van der Henst M. Poncin K. Francis N. et al. (2018). Transposon sequencing of brucella abortus uncovers essential genes for growth in vitro and inside macrophages. *Infect. Immun*. 86 e312–e318. 10.1128/IAI.00312-18 29844240 PMC6056880

[B31] Van der Henst C. de Barsy M. Zorreguieta A. Letesson J. J. De Bolle X. (2013). The brucella pathogens are polarized bacteria. *Microbes. Infect*. 15 998–1004. 10.1016/j.micinf.2013.10.008 24141086

[B32] Verbeke J. Fayt Y. Martin L. Yilmaz O. Sedzicki J. Reboul A. et al. (2023). Host cell egress of brucella abortus requires BNIP3L-mediated mitophagy. *EMBO J*. 42:e112817. 10.15252/embj.2022112817 37232029 PMC10350838

[B33] Wagner A. M. Eto H. Joseph A. Kohyama S. Haraszti T. Zamora R. A. et al. (2022). Dendrimersome synthetic cells harbor cell division machinery of bacteria. *Adv. Mater*. 34:e2202364. 10.1002/adma.202202364 35579491

[B34] Wang J. Moerner W. E. Shapiro L. (2021). A localized adaptor protein performs distinct functions at the caulobacter cell poles. *Proc. Natl. Acad. Sci. USA*. 118:e2024705118. 10.1073/pnas.2024705118 33753507 PMC8020655

[B35] Whatmore A. M. Foster J. T. (2021). Emerging diversity and ongoing expansion of the genus brucella. *Infect. Genet. Evol*. 92 104865. 10.1016/j.meegid.2021.104865 33872784

[B36] Yan Y. Wang Y. Yang X. Fang Y. Cheng G. Zou L. et al. (2022). The mincde cell division system participates in the regulation of type iii secretion system (T3ss) genes. *Microorganisms* 10:1549. 10.3390/microorganisms10081549 36013967 PMC9414521

[B37] Zhai Y. Fang J. Zheng W. Hao M. Chen J. Liu X. et al. (2024). A potential virulence factor: Brucella flagellin fliK does not affect the main biological properties but inhibits the inflammatory response in RAW264.7 cells. *Int. Immunopharmacol* 133:112119. 10.1016/j.intimp.2024.112119 38648715

[B38] Zhai Y. Wang H. Sun K. Yuan Y. Yin S. Fang J. et al. (2025). Enhancing host defense against brucella: The immune effect exerted by anti-OMP16 monoclonal antibody. *Int Immunopharmacol* 148:114142. 10.1016/j.intimp.2025.114142 39930647

