# Supplementary material for: Phosphodiesterase BpdE suppress bacterial proliferation by regulating min operon in Brucella abortus
Source: Front Microbiol. 2026 Aug 14;17:1899258. doi: 10.3389/fmicb.2026.1899258 (PMC13522176; doi:10.3389/fmicb.2026.1899258)
Supplement: Supplementary file 1 [file Data_Sheet_1.docx]

Supplementary Material

# 1 Supplementary Figures

## Figure S1. Construction and verification of the *bpdE* deletion in *B. abortus* A19.

(**A**) PCR amplification of the upstream and downstream homologous fragments of *bpdE*. Lane M: DNA marker; Lane 1: upstream fragment; Lane 2: downstream fragment. (**B**) Restriction enzyme digestion of the pUC19-SacB suicide plasmid. Lane M: DNA marker; Lane 1: undigested plasmid; Lane 2: plasmid digested with *Hind*III and *BamH*I. (**C**) PCR screening of the deletion strain using flanking primers. Lane M: DNA marker; Lane 1: WT control; Lanes 2 and 3: Δ*bpdE* candidates. (**D**) Verification of the *bpdE* deletion strain (Δ*bpdE*) by quantitative real-time PCR (qRT-PCR).

## Figure S2. Sanger sequencing verification of the Δ*bpdE* mutant.

**(A)** Schematic diagram showing the *bpdE* gene deletion strategy. **(B)** Sanger sequencing alignment confirming the successful and clean deletion of the *bpdE* in *B. abortus*. The alignment was generated using SnapGene software.

## Figure S3. Construction and verification of the *bpdE* complemented strain in *B. abortus* A19.

**(A)** PCR amplification of the full-length *bpdE* fragment. **(B)** Restriction enzyme digestion of the complementation vector (pBBR1-Amp-Flag). Lane 1: undigested plasmid; Lane 2: plasmid digested with *Hind*III. (**C**) Verification of the *bpdE* complemented strain (CΔ*bpdE*) by quantitative real-time PCR (qRT-PCR). (**D**) Western blot analysis confirming the expression of the BpdE protein (with a FLAG-tag) in CΔ*bpdE*. Omp16 was used as the internal loading control to ensure equal protein loading.

# 2 Supplementary Tables

**Supplementary Table 1.** Strains and plasmids listed in this study.

| **Strains/Plasmids** | **Description** | **Reference of Source** |
| --- | --- | --- |
| *Brucella abortus* A19 | Wild type strain | Lab stock |
| Δ*bpdE* | RS15270 knock-out fragment ligated on pUC-SacB | This study |
| CΔ*bpdE* | AmR; *bpdE* complete gene of *B.abortus* A19 | This study |
| Δ*bpdE*-*min*p-lacZ | Amp resistance gene, Δ*bpdE* carries a *bpdE*p-lacZ | This study |
| WT- *min*p-lacZ | Amp resistance gene, *B. abortus* A19 carries a *min* p-lacZ | This study |
| PUC19-SacB | Suicide vector; SacB, Amp resistance gene | Lab collection |
| pBBR1MCS-5 | Gentamicin resistance gene, mod, broad host range cloning vector | Lab collection |
| pBB-Amp-lacZ | Amp resistance gene, lacZ full fragment, expression vector, | Lab collection |
| pBB- *min*-p-lacZ | Amp resistance gene, *min* promoter, expression vector | This study |

**Supplementary Table 2.** PCR primers were used in this study.

| **Name** | **Primer sequence (5′-3′)** | **Usage** |
| --- | --- | --- |
| bpdE-up-F | cctgcaggcatgcaagcttCATTGCTCATATTGAGTATCGCCA | To amplify upstream fragment of *bbdP* in WT |
| bpdE-up-R | tccgggcggctggatttatgattgtcctgcacttt |  |
| bpdE-down-F | gcaggacaatcataaatccagccgcccggaaaac | To amplify downstream fragment of *bbdP* in WT |
| bpdE-down-R | accatgattacgccaagcttATTCGTTTGGCTGTATGCAAG |  |
| CΔbpdE-F | caggctctgggaggGAATTCaaaattcccagcccgcag | Used for CΔ*bbdP* construction |
| CΔbpdE-R | GTCATCCTTGTAATCAAGCTTtgcgtgcttttttttcctg |  |
| bpdE-I-F | CCACTTCTGCTGATGATCCG | Used for mutant selection and verification |
| bpdE-I-R | TTTCTGATCCTTCGCCGCTG |  |
| RT-bpdE-F | CATATGCCATTGAGCGCACC |  |
| RT-bpdE-R | AGCAGGCCGTCGTCAAAATA |  |
| RT-16S-F | ACGTGCTACAATGGTGGTGA |  |
| RT-16S-R | CAGAGTGCAATCCGAACTGA |  |
| RT-minC-F | AGATCCTCGGCGGTCTTGTA | Used for qRT-PCR |
| RT-minC-R | CGGTCTATTTCCCGGAAGGG |  |
| RT-minD-F | GATGATACCGAGCAGCGGAA |  |
| RT- minD-R | GTCGTCACCAATCCGGAAGT |  |
| RT-minE-F | TTTTCGCCGTCAGTGGAAGT |  |
| RT-minE-R | GTTTGCAGGTACTGCTTGCC |  |
| RT-dnaA-F | AGAGCCTGATGAAAATGGACAG |  |
| RT-dnaA-R | TGTTGATCCAGGAGCGCAAA |  |
| RT-ftsA-F | GCAAGGCCACTTGCATAAGG |  |
| RT-ftsA-R | TCACCTCGTTCATTCGCTCC |  |
| RT-ftsZ-F | ATGGAGGTTTCGTCGAGCAG |  |
| RT-ftsZ-R | CCACTTTTGCCGACGCTTTT |  |
| RT-ctrA-F | TGGATACGGGCAATCAGCTC |  |
| RT-ctrA-R | TGCTGGATCTCAATCTGCCG |  |
| RT-parA-F | ATGGTGGAACGCACCTGATT |  |
| RT-parA-R | ATTCTGGTGTTTCGGAGCGA |  |
| RT-parB-F | TTTCAACAGGCGCAATGTGT |  |
| RT-parB-R | GTTCGAGCTGATTGCTGGGG |  |
| min-F1 | gaaccgcagatttcgcaagc | Used for reverse transcription-PCR |
| min-R1 | atgacaagatccacgccctc |  |
| min-F2 | gacatgctgaaggtcgagga |  |
| min-R2 | atatcgacctccagcgtcga |  |
| min-F-lacZ | GTGGTGGTGCTCGAGcttggttaacggcaagttaa | Used for β-galactosidase activity assay |
| min-R-lacZ | GCTGATATCGGATCCgatggcggaagtatagcaac |  |
